# Supplementary material for: Liaison psychiatry—measurement and evaluation of service types, referral patterns and outcomes (LP-MAESTRO): a protocol
Source: BMJ Open. 2019 Nov 24;9(11):e032179. doi: 10.1136/bmjopen-2019-032179 (PMC6887012; doi:10.1136/bmjopen-2019-032179)
Supplement: Author's manuscript [file bmjopen-2019-032179.draft_revisions.pdf]

BMJ Open is committed to open peer review. As part of this commitment we make the peer review history of every article we publish publicly available.

When an article is published we post the peer reviewers' comments and the authors' responses online. We also post the versions of the paper that were used during peer review. These are the versions that the peer review comments apply to.

The versions of the paper that follow are the versions that were submitted during the peer review process. They are not the versions of record or the final published versions. They should not be cited or distributed as the published version of this manuscript.

BMJ Open is an open access journal and the full, final, typeset and author-corrected version of record of the manuscript is available on our site with no access controls, subscription charges or pay-per-view fees (http://bmjopen.bmj.com).

If you have any questions on BMJ Open's open peer review process please email info.bmjopen@bmj.com

BMJ Open

Liaison Psychiatry - Measurement and Evaluation of Service Types, Referral patterns and Outcomes - LP- MAESTRO: a protocol

Journal:	*BMJ Open*
Manuscript ID	bmjopen-2019-032179
Article Type:	Protocol
Date Submitted by the Author:	06-Jun-2019

Complete List of Authors:	Smith, Chris; University of Leeds, Leeds Institute of Health Sciences<br>Hewison, Jenny; University of Leeds, Leeds Institute of Health Sciences<br>West, Robert; University of Leeds, Leeds Institute of Health Sciences<br>Guthrie, Elspeth; University of Leeds, Leeds Institute of Health Sciences<br>Trigwell, Peter; Leeds and York Partnership NHS Foundation Trust, National Inpatient Centre for Psychological Medicine<br>Crawford, Mike; Imperial College London, Centre for Psychiatry, Department of Medicine; Royal College of Psychiatrists, College Centre for Quality Improvement<br>Czoski Murray, Carolyn; University of Leeds, Leeds Institute of Health Sciences<br>Fossey, Matt; Anglia Ruskin University, Veterans and Families Institute for Military Research, Faculty of Health, Social Care and Education<br>Hulme, Claire; University of Exeter, University of Exeter Medical School<br>Tubeuf, Sandy; Universite catholique de Louvain<br>House, Allan; University of Leeds, Leeds Institute of Health Sciences

Keywords:	routinely-collected NHS data, data linkage, care pathways, liaison psychiatry

SCHOLARONE™
Manuscripts

Liaison Psychiatry - Measurement and Evaluation of Service Types, Referral patterns and Outcomes - LP- MAESTRO: a protocol

Authors

Chris Smith (C.J.Smith@leeds.ac.uk)
Leeds Institute of Health Sciences (LIHS), University of Leeds, Leeds, UK

Jenny Hewison (J.Hewison@leeds.ac.uk)
Leeds Institute of Health Sciences (LIHS), University of Leeds, Leeds, UK

Robert M West (R.M.West@leeds.ac.uk)
Leeds Institute of Health Sciences (LIHS), University of Leeds, Leeds, UK

Elspeth Guthrie (E.A.Guthrie@leeds.ac.uk)
Leeds Institute of Health Sciences (LIHS), University of Leeds, Leeds, UK

Peter Trigwell (peter.trigwell@nhs.net)
National Inpatient Centre for Psychological Medicine, Leeds and York Partnership NHS Foundation Trust, Leeds, UK

Mike J Crawford (m.crawford@imperial.ac.uk)
College Centre for Quality Improvement, Royal College of Psychiatrists, London, UK
Centre for Psychiatry, Department of Medicine, Imperial College London, London, UK

Carolyn Czoski-Murray (C.J.CzoskiMurray@leeds.ac.uk)
Leeds Institute of Health Sciences (LIHS), University of Leeds, Leeds, UK

Matt Fossey (matt.fossey@anglia.ac.uk)
Veterans and Families Institute for Military Research, Faculty of Health, Social Care and Education, Anglia Ruskin University, Chelmsford, UK

Claire Hulme (C.T.Hulme@exeter.ac.uk)
University of Exeter Medical School, University of Exeter, Exeter, UK

Sandy Tubeuf (sandy.tubeuf@uclouvain.be)
Institute of Health and Society, Université Catholique de Louvain, Louvain-la-Neuve, Belgium

Allan House (A.O.House@leeds.ac.uk)
Leeds Institute of Health Sciences (LIHS), University of Leeds, Leeds, UK

Corresponding Author

Chris Smith (C.J.Smith@leeds.ac.uk)
Leeds Institute of Health Sciences (LIHS), University of Leeds, Leeds, UK

Liaison Psychiatry - Measurement and Evaluation of Service Types, Referral patterns and Outcomes - LP- MAESTRO: a protocol

Abstract

Background

We describe the protocol for a project that will use linkage of routinely-collected NHS data to answer a question about the nature and effectiveness of liaison psychiatry services in acute hospitals in England.

Methods

The project will use three data sources: i) Hospital Episode Statistics (HES), a database controlled by NHS Digital that contains patient data relating to emergency department, inpatient and outpatient episodes at hospitals in England; ii) ResearchOne, a research database controlled by The Phoenix Partnership (TPP) that contains patient data relating to primary care provided by organisations using the SystmOne clinical information system; and iii) clinical databases controlled by mental health trusts that contain patient data relating to care provided by liaison psychiatry services. We will link patient data from these sources to construct care pathways for patients who have been admitted to a particular hospital and determine those patients that have been seen by a liaison psychiatry service during their admission.

The patient care pathways will form the basis of a matched cohort design to test the effectiveness of liaison intervention. We will combine health care utilisation within care pathways using cost figures from national databases. We will compare the cost of each care pathway and the impact of a broad set of health-related outcomes to obtain preliminary estimates of cost-effectiveness for liaison psychiatry services. We will carry out an exploratory incremental cost-effectiveness analysis from a whole system perspective.

Discussion

Studies based on the linkage of routinely-collected NHS data enable the generation of evidence regarding the cost-effectiveness and efficiency of health services, where alternative study designs such as RCTs or other individually-consented study designs would be infeasible. Studies such as this present technical, ethical and legal challenges. Study designs should consider such challenges from the start, including their implications for validity, generalisability and project resources.

Keywords

Liaison psychiatry, routinely-collected NHS data, data linkage, care pathways

Strengths and Limitations of this study

- Study designs based on the linkage of routinely-collected NHS data enable the generation of evidence regarding the cost-effectiveness and efficiency of health services, where alternative study designs such as RCTs or other individually-consented study designs would be infeasible.

- Whilst our study will evaluate the impact of acute inpatient hospital work carried out by liaison teams, and will not cover work undertaken in the Emergency Department, outpatient or primary care setting, the findings will be highly relevant, as previous claims for cost savings resulting from the implementation of liaison services have been primarily based on their inpatient hospital work.

- Study designs based on the linkage of routinely-collected NHS data present technical, ethical and legal challenges which must be considered from the start, including their implications for the validity and generalisability of insights and for project resources.

Background

Liaison psychiatry services provide assessment and treatment for people with co-existent physical and mental health problems (1-4). Such services are provided predominantly in the acute hospital setting in the United Kingdom, although more recently services have emerged to support the management of people with complex physical and mental health problems in primary as well as secondary care (5). Liaison psychiatry services have the potential to improve both the quality of care and overall outcomes for people with mental and physical health problems. There is also a suggestion that liaison psychiatry services in the acute hospital setting will produce cost savings by reducing length of stay, even though it is estimated that services see a small proportion (1-5%) of all patients admitted to acute beds (6-12). For these reasons, NHS England has invested in expanding liaison psychiatry services to acute hospitals (13, 14).

The research evidence has not been strong for the cost-effectiveness of liaison psychiatry *services* (15, 16) as opposed to evidence on the cost-effectiveness of some of the *individual interventions* used in those services. (17, 18). For that reason, more research is needed using robust designs derived from health services research. Claims for cost-effectiveness of liaison psychiatry go to the origins of the specialty before World War II. Individual components have been tested in Randomised Controlled Trials (RCTs), but there have been few attempts to judge the cost-effectiveness of whole services. Although it has been argued they can pay for themselves - the cost-offset debate of the 1980 and 1990s (19) - in truth the cost-effectiveness evidence for any liaison psychiatry service is limited. Holmes et al (20) identified only two RCTs, some smaller non-randomised studies include working age and older patients, and older and non-definitive work on cost-offsetting.

There are three main reasons why visiting the question now is timely.

First, cost pressures in the NHS and the emergence of commissioning led by Clinical Commissioning Groups (CCGs) will continue to lead to re-profiling of services and especially attempts to reduce unnecessary hospital costs. Work on developing and implementing risk stratification models are an illustration and it is interesting that many of these models identify co-morbid physical and mental health problems as a risk, both for increased healthcare costs and for the main driver of such costs which is unscheduled hospital admission. A study from Birmingham describing the evaluation of a rapid assessment, intervention and discharge service (previously widely known by the acronym RAID) was widely promoted and is leading to commissioning of similar services that will be hoped to reduce costs (21, 22). There were however substantial problems with that evaluation: it reported only on the first 9 months of delivery of a new service; it used a simple before and after design; it compared outcomes between referred and a matched group of non-referred patients in only 79 cases with minimal matching that cannot have dealt with indication bias; and much of the benefit was attributed to so-called indirect liaison cases who were not in fact seen by the service but assumed to benefit

by its existence in the hospital. Other 'RAID'-like services have also reported large savings in cost or reductions in hospital use following implementation (23, 24). So a key answer to the question of why this research is needed now is the pressing need to confirm or refute the very striking claims made for similar services, but using larger numbers and more robust research methods.

Second, there is a danger of losing sight of the other main functions of liaison psychiatry services, which don't exist only to reduce costs in the general hospital but which are aimed at improving the wellbeing of patients, some of whom are being treated entirely appropriately in the general hospital and happen to need mental health service input because of the complexity of their problems.

Third, and related to the second point above, service commissioning and provision will benefit from a more standardised approach to service descriptors. Without more detailed knowledge about how to define the service being commissioned and how to evaluate whether it is working to remit and to improve Health-Related Quality of Life (HRQL) for patients, there is a risk of enthusiastic commissioning of services that look superficially similar to each other and to a model reported as cost-effective, when in fact either at the time or over a period after commissioning there are differences in staffing or referral patterns that invalidate the original commissioning assumptions. An important function is served by studies that describe what patient groups actually receive - what sort of service and in what numbers.

This research is thus timely in exploring methods to evaluate the function and performance of liaison psychiatry services.

There are, however, challenges in conducting such research.

First, defining exposure to liaison psychiatry is difficult because there is substantial heterogeneity in the composition, purpose and size of liaison psychiatry services. For example, a recent survey conducted in England (25) found that just over half of the services provided a 24 hour 7-day service and only a third ran specialist outpatient clinics. Most of the services provided cover of acute hospital wards and emergency department and nearly all services were multidisciplinary, but staff mix varied such that about a third employed less than one fulltime equivalent of a consultant in psychiatry. Only a third of services had separate teams for older adults and adults of working age. These differences were not fully explained by variation in acute hospital characteristics. Also, the mechanism by which liaison psychiatry services might produce improved patient and organisational outcomes is unclear – for example, with some suggesting that the main benefit is by securing rapid discharge to community-based services, while others emphasise the specialist nature of shared in-patient management or of outpatient clinics provided by the service.

Second, defining service outcomes is difficult because there is also substantial heterogeneity in the patient populations seen in liaison services. For example, service outcomes (and performance indicators) will not be the same for somebody seen in the emergency department after an act of self-harm, an older person with post-operative agitated behavior seen on a surgical ward, or somebody with chronic unexplained pain referred from a pain clinic.

To evaluate the impact of liaison psychiatry services on the outcomes of patients in acute hospital settings, we therefore need to be able to do three things. *Patients* attending selected hospitals need to be characterized with respect to their physical and mental health. The prognosis for any given mental health problem is strongly influenced by the prior history of mental disorder, so the nature of the psychiatric problem needs to be described not just at the time of admission but in the preceding months. This longitudinal picture can only be determined reliably and for all patients in any sample by the use of routine data from primary care. Patient *healthcare contact* in both primary and secondary care services needs to be recorded. And *outcomes* need to be identified beyond the immediate spell in hospital. The heterogeneity of both patient population and service exposures requires a large study in terms of number of hospitals and patients.

In addition to these measurement problems, there is a challenge in choosing a robust research design. There have been several RCTs showing the effectiveness of individual components of liaison services, but an individually-randomised RCT of a whole service configuration would be impractical. The *heterogeneity of case mix* even in simpler services will require numbers beyond what could be reasonably recruited, and there are major challenges in obtaining a large representative sample when individual consent is required. Because many patients seen by liaison psychiatry services lack mental capacity at the time of service contact, an individually-consented study is not feasible and an individually-randomised RCT would not in any case answer the service-level question.

We also considered a cluster RCT of different liaison services. *Heterogeneity of service provision*, as identified in our national survey of services in England, would make such a study prohibitively large.

For these reasons, our view is that a large-scale observational study based upon analysis of *routinely-collected NHS data* and which is not predicated on individual patient consent is the best option.

Methods

Study design

Retrospective cohort design, comparing outcomes for patients admitted to hospital and seen by a liaison psychiatry service with two control groups – the first is a patient group who were admitted to the same

hospital in the same study period and matched on hospital inpatient and primary care data, but were not seen by the liaison psychiatry service. Because such a design could not entirely exclude confounding by indication, we will use a second matched patient group who had been admitted to a different hospital without a liaison psychiatry service in the same study period. This second group will also be matched using data from hospital and primary care records; however they will not have been selected on the basis of whether the responsible (acute hospital) consultant had made a decision about liaison psychiatry referral.

Aims and objectives

The aim of the study described in this paper is to examine care pathways for the main target populations of liaison psychiatry services and estimate the outcomes and costs associated with care. Specifically, we will:

1. Use routinely-collected NHS data to identify patients referred to study services and matched comparison patients who were not referred, with the aim of comparing within and between hospitals the effect of referral or non-referral of patients with similar characteristics.

2. Estimate the cost of the care pathways of patients referred to liaison psychiatry services, and the matched comparison patient groups, and the main determinants of those costs over 12 months after an index hospital episode.

We will characterise patients according to their contact with liaison psychiatry services: referrals to the liaison psychiatry service from acute (general hospital) sources and a matched sample of cases from the same sources who were not referred. We will compare outcomes for certain marker conditions in different liaison service configurations.

This study represents one component (Work Stream 2) of a wider project, LP MAESTRO (25), designed to evaluate the cost-effectiveness and efficiency of particular configurations of liaison psychiatry service.

Data sources

Patient data that are relevant to the study are routinely-collected by clinicians and healthcare professionals to inform patient care. Such data are collected independently by the organisations that provide different services to patients, and only those variables that are required to fulfil the purpose(s) of these services are included. A limited number of standardised datasets are collected from organisations that provide care and are aggregated at national-level by organisations, such as NHS Digital. Some of these datasets (or derivatives) are made available for research purposes. However, no single organisation can currently provide the data that is required for the study, so linkage is essential.

Hospital Episode Statistics (HES) (27) is a database controlled by NHS Digital that contains patient data relating to emergency department, inpatient and outpatient episodes for NHS hospitals in England. Episodes represent discrete periods of care under a particular consultant. Episodes can be combined into spells to represent the period of care from admission to discharge. HES is derived from the Commissioning Data Set (CDS) (28), which is supplied to NHS Digital by organisations that provide NHS services to facilitate monitoring and payment. Patients can opt-out of the inclusion of their *confidential patient information* in datasets which are made available by NHS Digital for purposes beyond care, such as HES, through the national opt-out programme (29). HES is an important source of data relating to health service interaction in secondary care. There are three significant limitations that are relevant to this study.

Firstly, referral to liaison psychiatry services cannot be reliably determined from HES. A new episode is not generated by such a referral - the patient remains within the care of the acute hospital consultant - and contact with a different consultant-led team in liaison psychiatry is not represented within an episode.

Secondly, it is suggested that mental health diagnoses recorded in routinely-collected NHS data, such as HES, exhibit variable accuracy with respect to the true diagnosis (30).

Thirdly, patient interactions with primary care services are not included in HES. Such data are required to match patients by defined characteristics and to determine the care delivered in primary care following a general hospital admission that leads to a liaison psychiatry referral.

To address the first limitation, clinical databases controlled by the mental health trusts that provide liaison psychiatry services will be used. Such databases contain patient data relating to care provided by liaison psychiatry services and can be used in conjunction with HES data to determine whether a patient was referred to a liaison psychiatry service during a hospital admission. The main challenges with the use of such databases are data quality and the heterogeneous processes by which data access is negotiated and administered within different organisations.

To address the second and third limitation, data relating to primary care is required for each patient. Although the Clinical Practice Research Datalink (CPRD) database (31) is widely used for primary care research in the United Kingdom, it has a major drawback for this study – limited numbers and geographical coverage of participating primary care organisations at the time of study design. Instead ResearchOne (32), a research database controlled by The Phoenix Partnership will be used. ResearchOne contains patient data relating to primary care provided by organisations using the SystmOne clinical information system (33). SystmOne (34%) and EMIS (34) (56%) are the most prevalent clinical information systems used by organisations in general practice (35). Therefore, many of the patients with episodes in the HES data can be expected to have data relating to their primary care collected by organisations that use SystmOne.

ResearchOne contains data for a subset of these patients – patients who: i) are registered to organisations that use SystmOne and have opted-in to participation in ResearchOne, and ii) have not individually opted-out of participation in ResearchOne. The main challenge with the use of ResearchOne is the inability to determine *a priori* the numbers and geographical coverage of organisations that provide primary care to patients attending the hospitals chosen for the study, and the resultant ability to match HES data to corresponding ResearchOne data for each patient.

Patient data from these three sources will be linked to construct patient care pathways that span primary and secondary care settings. Linkage will be undertaken by NHS Digital. Each data source will generate two unique references for each patient: i) a pseudonym - generated by applying a one-way cryptographic hash function (SHA-512) to an input that comprises a cryptographic salt and their NHS number, and ii) a source-specific identifier. For a patient with a given NHS number, each data source will generate the same pseudonym but a different source-specific identifier. Both the pseudonym and source-specific identifier generated for each patient will be specific to the study. Pseudonyms will be used by NHS Digital to: i) communicate to data sources those patients for whom data is required, and ii) generate mappings between the different source-specific identifiers for each patient. Data sources will provide the required patient data to the research team, including only the source-specific identifier as the unique reference for each patient. The mappings generated by NHS Digital will be provided to the research team and used to determine the data that relates to each patient across the data received from the different sources.

Data extraction

Based on the results from earlier stages of the LP MAESTRO project, we will identify at least two and up to six configurations that best represent patterns of liaison psychiatry service across England. We will sample purposively to obtain 2-4 services of each type (depending upon availability). Data will be extracted for patients attending the hospitals with these services in the index period (financial year 2013/14) and also for patients attending hospitals identified as not having a liaison psychiatry service in the same period.

Relevant variables extracted for each patient from the sources will include demographic variables (e.g. age, carer support, Index of Multiple Deprivation – a measure of locality deprivation), clinical variables (e.g. diagnosis, medications) and health service utilisation variables (e.g. inpatient days, GP appointments, major procedures). One of the novelties of our approach is the use of variables obtained from primary and secondary care settings to tackle the substantial challenge that comes from indication bias; for example we will use variables obtained from ResearchOne to define healthcare utilisation in primary care for the year before referral (2013), as a way of ensuring that outcomes in the year after referral (2015) are not attributed to easily identifiable pre-existing characteristics (case complexity) that are confounded with likelihood of referral.

Patient care pathways for patients attending hospitals using liaison psychiatry services in each configuration will be constructed to provide a view of health and healthcare across both primary and secondary care. Pathways will be constructed for patients for a period of 12 months following their index (first) hospital admission in the index period. The cost of each pathway to the health care sector will then be calculated using national data sources (see below). We will adopt a whole system perspective in order to determine if there is an association between the configuration of liaison psychiatry services and health care utilisation by patients. Metrics including emergency admissions, occupied bed days and length of stay will be analysed by age band.

Data analysis

We will build a standard regression model to estimate the relation between health care utilisation and key variables capturing the configuration of liaison psychiatry services. The dependent variable in this model will therefore be the total costs of health care utilisation derived from factors such as inpatient days, readmission rates, ED attendances and GP visits combined with reference costs. Costs will be valued using national sources, where possible including the Department of Health Reference Costs (36) and Personal Social Services Research Institute (PSSRU) Costs for Health and Social Care. (37). Where these are not available, local costs will be assigned. The quantum of the liaison service provision will be captured by already collected data related to structure and process; for example, staffing levels and contact time after referral. We will adjust for referral indication bias, either by matching for co-variates or by propensity scoring.

Sample size is difficult to estimate because we have so little available data on outcomes for different service types and different patient groups. Suppose we identify six main service configurations and recruit two liaison services for each (total n=12). For less common conditions we might expect to see one referral per week per service = 100 in total in the year. For more common conditions we might perhaps see one referral per day or 600-800 in the year. These numbers will allow us to estimate the costs and cost-effectiveness of liaison services with substantially greater precision than has been achieved to date – for example by the RAID evaluation.

The way in which components of general hospital, general mental health and liaison services interact with each other is complex and a key part of the project will be to determine how to capture this complexity into a set of measures for inclusion in the model.

We will carry out exploratory incremental cost effectiveness analyses using economic evaluation techniques and decision analysis modelling. The model will rely on the retrospectively estimated healthcare costs and the characteristics at hospital, service and patient-level. Given the nature of the data available, the absence

For peer review only - http://bmjopen.bmj.com/site/about/guidelines.xhtml

of measures such as Quality of Life measures and the heterogeneous nature of the population, we will explore the use of a range of variables including length of stay, readmission and mortality to assess effectiveness. Thus the analyses will be informed by earlier work packages in the LP-MAESTRO project. We will however also follow the guidance from the National Institute for Clinical Excellence (NICE) for methods for technology appraisal (38). We will adopt a whole system perspective and compare cost and effectiveness of the "referred" group over the matched group. However, whilst it is clear that some aspects of an exploratory model (or indeed models) may be specified in advance, for example, the perspective of the economic evaluation which will be the health service provider and the comparator which will be usual care, other aspects will be dependent upon the shape of the services and the populations they engage with.

At this point we are unable to specify the time horizon of the decision analysis model evaluating the long-term cost-effectiveness of liaison services. We will look to a long-term model and use NICE recommended discount rates for costs and outcomes. The model itself is likely to be Markov or semi-Markov. Sensitivity analyses will be undertaken in line with those recommended for this type of modelling (39). Presentation of the analysis or analyses will include incremental cost-effectiveness ratios (ICERs), cost-effectiveness acceptability curves and net monetary benefit estimates. In addition, we will undertake a value of information (VOI) analysis (40).

Patient and Public Involvement

The study has a Patient and Public Involvement (PPI) representative on the Study Steering Committee that oversees the management of the research.

Ethics and governance

The study is funded by the National Institute of Health Research under the Health Service and Delivery Research programme (REF: 13/58/08) and is sponsored by the University of Leeds. The study is based at the Leeds Institute of Health Sciences within the University of Leeds and will use the Information Governance Toolkit (IGT) compliant infrastructure at the Leeds Institute of Clinical Trials Research (REF: ECC0010).

To simplify the documentation provided to underpin ethics and governance processes, the study has been partitioned into distinct phases. Each phase is characterized by the use of a specific combination of data sources to construct care pathways for patients attending a particular hospital or set of hospitals. Phase 1 is characterized by the use of data from HES and ResearchOne *only* to construct care pathways for patients attending hospitals *without* a liaison psychiatry service. A summary of the ethics and governance processes undertaken for Phase 1 is provided below.

Phase 1 was submitted to the NHS Research Ethics Committee (North of Scotland) on 23rd February 2016 (REF: 16/NS/0025). The application was reviewed in a meeting held on the 10th March 2016 and received a favorable ethical opinion on 15th March 2016. Favorable ethical opinion was contingent on obtaining management permission from the hospitals whose patients were to be included in the HES data. Management permission was received for 8 of the 11 hospitals by a stated deadline (15th December 2016) and Phase 1 proceeded based on the use of HES data from these 8 hospitals only.

Phase 1 was also submitted to the Confidentiality Advisory Group (CAG) (41) at the Health Research Authority on 23rd February 2016 (REF: 16/CAG/0037) to determine whether the study required Section 251 support under Regulation 5 of the Health Service (Control of Patient Information) Regulations 2002 (42). The application was reviewed in a meeting on 21st April 2016 and was deferred pending the receipt of further information in relation to the linkage process from the research team. Further information was supplied and CAG provided a decision on 19th July 2016 that Section 251 support was not required "*on the basis that there is no disclosure of patient identifiable data without consent*".

Based on favorable ethical opinion and a decision from CAG that Section 251 support was not required, approval in principle for Phase 1 was provided by TPP on 11th October 2016. Data requests were submitted to NHS Digital on 16th December 2016 (REF: NIC-77953) and TPP on 22nd February 2017. Supporting evidence documents were provided with these requests, which included confirmation of favorable ethical opinion, confirmation of the CAG decision on Section 251 support, and details of the technical and organisational safeguards in place at the data controller and processors. Organisational safeguards included a Data Processing Agreement established between University of Leeds and TPP to cover the data processing activities within Phase 1. The application was reviewed by the Independent Group Advising on the Release of Data (IGARD) (43) at NHS Digital on 20th March 2018. Further information was requested from the project team by IGARD, which was subsequently supplied, and a recommendation to approve the application was provided in a meeting on 26th April 2018. A Data Sharing Agreement (DSA) was established between the University of Leeds and NHS Digital for Phase 1 on 26th April 2018, which was underpinned by the pre-existing Data Sharing Framework Contact between the University of Leeds and NHS Digital (REF: CON-315426-K3W7R).

Data were supplied by NHS Digital on 16th November 2018 and the remaining data from NHS Digital and TPP are currently awaited.

Discussion

Studies based on the linkage of routinely-collected NHS data enable the generation of evidence regarding the cost-effectiveness and efficiency of health services, where alternative study designs such as RCTs or other

individually-consented study designs would be infeasible. Health service commissioners can be provided with robust evidence to underpin decisions regarding health services and interventions – in this case relating to the cost-effectiveness and efficiency of different liaison psychiatry configurations for specified target populations – where previously there may have been limited or no evidence. The findings of this study will evaluate the impact of acute inpatient hospital work carried out by liaison teams, and does not cover work undertaken in the Emergency Department, outpatient or primary care setting. The findings, however, will be highly relevant, as previous claims for cost savings resulting from the implementation of liaison services have been primarily based on their inpatient hospital work.

Studies such as this present technical, ethical and legal challenges. Study designs should consider such challenges from the start, including their implications for the validity and generalisability of insights and for project resources. Experience from LP-MAESTRO demonstrates that significant resources are required to design and communicate a research protocol in the manner that satisfies the different project stakeholders, including research ethics committees, data controllers, regulatory bodies and data access committees. Different stakeholders are focused on their specific remit and require communication of the research protocol in accordance with that remit. Moreover, different stakeholders may comprise of decision-makers from different disciplines and require the research protocol to be communicated at differing levels of abstraction to ensure adequate comprehension. This issue of communication between disciplines and the potential for misinterpretation is highlighted in a recent Nuffield foundation report (44).

The project described here is both technically feasible and consistent with current legislative and ethical frameworks applicable to the use of health data for research purposes. The main practical challenges reside in the communication with, negotiation between, and coordination of different stakeholders as outlined above.

List of Abbreviations

RCTs: Randomised Controlled Trials
CCGs: Clinical Commissioning Groups
HRQL: Health-Related Quality of Life
HES: Hospital Episode Statistics
CDS: Commissioning Data Set
CPRD: Clinical Practice Research Datalink
PSSRU: Personal Social Services Research Institute
NICE: National Institute for Clinical Excellence
VOI: Value of Information
IGT: Information Governance Toolkit
CAG: Confidentiality Advisory Group
IGARD: Independent Group Advising on the Release of Data
DSA: Data Sharing Agreement
TPP: The Phoenix Partnership

Declarations

Ethics approval and consent to participate

Favourable ethical opinion has been obtained from the NHS Research Ethics Committee (North of Scotland) (REF: 16/NS/0025) for Work Stream 2 (Phase 1) of the LP-MAESTRO study.

Individual patient consent will not be feasible for this study. Favourable ethical opinion was obtained from the NHS Research Ethics Committee (North of Scotland) (REF: 16/NS/0025) for a study protocol where individual patient consent was not obtained. The Confidentiality Advisory Group at the Health Research Authority determined that Section 251 approval under Regulation 5 of the Health Service (Control of Patient Information) Regulations 2002 was not required for the study "*on the basis that there is no disclosure of patient identifiable data without consent*" (REF: 16/CAG/0037).

Consent for publication

Not applicable.

Availability of data and material

Not applicable.

Competing interests

CS is Director of PrivacyForge Limited. All other authors declare that they have no competing interests.

Funding

This project is funded by the National Institute for Health Research (NIHR) Health Services and Delivery Research (HS&DR) programme (project reference 13/58/08).

Authors' contributions

CS, JH, RW, EG, PT, MC, CCM, MF, CH, ST, and AH contributed to the design of the study protocol. AH, JH and CS authored the manuscript. CS, JH, RW, EG, PT, MC, CCM, MF, CH, ST, and AH contributed to the revision of the manuscript and approved the final version.

Acknowledgements

The views expressed are those of the author(s) and not necessarily those of the National Institute for Health Research (NIHR) or the Department of Health and Social Care.

References

1. Aitken P, Lloyd G, Mayou R, Bass C, Sharpe M. A history of liaison psychiatry in the UK. BJPsych Bull. 2016;40(4): 199-203.
2. Lipowski Z. Consultation-liaison psychiatry: the first half century. General hospital psychiatry. 1986;8(5): 305-15.
3. Mayou R. The history of general hospital psychiatry. Br J Psychiatry. 1989;155: 764-76.
4. Schwab JJ. Consultation-liaison psychiatry: a historical overview. Psychosomatics. 1989;30(3): 245-54.
5. Bestall J, Siddiqi N, Heywood-Everett S, Freeman C, Carder P, James M, et al. New models of care: a liaison psychiatry service for medically unexplained symptoms and frequent attenders in primary care. The Psychiatrist: Bulletin of the Royal College of Psychiatrists. 2017;41(6): 340-4.
6. Huyse, F.J., et al. European consultation-liaison services and their user populations: the European Consultation-Liaison Workgroup Collaborative Study. Psychosomatics. 2000;41(4): 330-8.
7. Diefenbacher, A., Implementation of a psychiatric consultation service: a single-site observational study over a 1-year-period. Psychosomatics. 2001;42(5): 404-10.
8. Christodoulou, C., et al. Transfers to psychiatry through the consultation-liaison psychiatry service: 11 years of experience. Ann Gen Psychiatry, 2008. 7.
9. Su, J.A., et al. Changes in consultation-liaison psychiatry in the first five years of opearation of a newly-opened hospital. Chang Gung Med J. 2010;33(3): 292-300.
10. Sanchez-Gonzalez, R., et al. Clinical features of a sample of inpatients with adjustment disorder referred to a consultation-liaison psychiatry service over 10years. Gen Hosp Psychiatry. 2018;55: 98-99.
11. Sanchez-Gonzalez, R., et al. Clinical profile of inpatients referred to a consultation-liaison psychiatry service: an observational study assessing changes over a 10-yearperiod. Actas Esp Psiquiatr, 2018;46(2): 58-67.
12. Gala, C., et al. A multicenter investigation of consultation-liaison psychiatry in Italy. Gen Hosp Psychiatry. 1999;21(4): 310-7.
13. NHS England. Five Year Forward View. London: NHS England; 2014.
14. NHS England. Urgent and emergency mental health liaison in acute hospitals (adult and older adults): Supporting documentation for transformation funding selection. London: NHS England; 2016.
15. de Araujo Andreoli PB, de Albuquerque Citero V, de Jesus Mari JJP. A systematic review of studies of the cost-effectiveness of mental health consultation-liaison interventions in general hospitals. Psychosomatics. 2003;44(6): 499-507.
16. Hosaka T, Aoki T, Watanabe T, Okuyama T, Kurosawa HJP. General hospital psychiatry from the perspective of medical economics. Psychiatry and Clinical Neurosciences. 1999;53(4): 449-53.

17. Ruddy R, House A. Meta-review of high-quality systematic reviews of interventions in key areas of liaison psychiatry. Br J Psychiatry. 2005;187: 109-20.
18. Wood R, Wand APF. The effectiveness of consultation-liaison psychiatry in the general hospital setting: A systematic review. Journal of Psychosomatic Research. 2014;76(3): 175-92.
19. Strain JJ, Hammer JS, Fulop G. APM task force on psychosocial interventions in the general hospital inpatient setting: a review of cost-offset studies. Psychosomatics. 1994;35(3) :253-62.
20. Holmes A, Handrinos D, Theologus E, Salzberg M. Service use in consultation-liaison psychiatry: guidelines for baseline staffing. Australasian Psychiatry. 2011;19(3): 254-8.
21. Parsonage M, Fossey M. Economic Evaluation of a Liaison Psychiatry Service. London: Centre for Mental Health; 2011.
22. Tadros G SR, Kingston P, Mustafa N, Johnson E, Pannell R, Hashmi M. Impact of an integrated rapid response psychiatric liaison team on quality improvement and cost savings: the Birmingham RAID model. The Psychiatrist. 2013;37: 4-10.
23. Becker L, S.R., Hardy R, Pilling S. The RAID Model of Liaison Psychiatry: Report on the Evaluation of Four Pilot Services in East London. London: UCL Partners Academic Health Science Partnership; 2016.
24. Opmeer, B.C., et al., Extending the liaison psychiatry service in a large hospital in the UK: a before and after evaluation of the economic impact and patient care following ED attendances for self-harm. BMJ Open. 2017;7(8).
25. Walker A, Barrett JR, Lee W, West RM, Guthrie E, Trigwell P, et al. Organisation and delivery of liaison psychiatry services in general hospitals in England: results of a national survey. BMJ Open. 2018;8(8).
26. National Institute for Health Research (NIHR). Liaison Psychiatry: Measurement And Evaluation of Service Types, Referral patterns and Outcomes (LP-MAESTRO). https://www.journalslibrary.nihr.ac.uk/programmes/hsdr/135808 (2019). Accessed 19th Feb 2019.
27. NHS Digital. Hospital Episode Statistics (HES). https://digital.nhs.uk/data-and-information/data-tools-and-services/data-services/hospital-episode-statistics (2019). Accessed 19th Feb 2019.
28. NHS Digital. Commissioning Datasets. https://digital.nhs.uk/data-and-information/data-collections-and-data-sets/data-sets/commissioning-data-sets (2019). Accessed 11th Apr 2019.
29. NHS Digital. National Opt-Out Programme. https://digital.nhs.uk/services/national-data-opt-out-programme (2019). Accessed 19th Feb 2019.
30. Davis KA, Sudlow CL, Hotopf MJBp. Can mental health diagnoses in administrative data be used for research? A systematic review of the accuracy of routinely collected diagnoses. BMC Psychiatry. 2016;16(1):263.
31. Clinical Practice Research Datalink. https://www.cprd.com (2019). Accessed 19th Feb 2019.
32. The Phoenix Partnership. ResearchOne. http://www.researchone.org (2019). Accessed 19th Feb 2019.

33. The Phoenix Partnership. SystmOne. https://www.tpp-uk.com/products/systmone (2019). Accessed 19th Feb 2019.
34. Egton Medical Information Systems. EMIS Web. https://www.emishealth.com/products/emis-web/ (2019). Accessed 19th Feb 2019.
35. Kontopantelis E, Stevens RJ, Helms PJ, Edwards D, Doran T, Ashcroft DMJBo. Spatial distribution of clinical computer systems in primary care in England in 2016 and implications for primary care electronic medical record databases: a cross-sectional population study. BMJ Open. 2018;8(2).
36. Department of Health. Reference costs 2015–16. London, UK: NHS England, 2016.
37. Curtis, L. & Burns, A. (2018) Unit Costs of Health and Social Care 2018, Personal Social Services Research Unit, University of Kent, Canterbury.
38. National Institute for Health and Care Excellence. Guide to the methods of technology appraisal. London: NICE, 2013.
39. Briggs R, Sculpher M, Claxton K. Decision Modelling for Health Economic Evaluation. Oxford, UK: Oxford University Press; 2006.
40. Petrou S, Gray A. Economic evaluation using decision analytical modelling: decision, conduct, analysis, and reporting. BMJ 2011;342.
41. Health Research Authority. Confidentiality Advisory Group. https://www.hra.nhs.uk/approvals-amendments/what-approvals-do-i-need/confidentiality-advisory-group (2019). Accessed 19th Feb 2019.
42. HM Government. The Health Service (Control of Patient Information) Regulations 2002. http://www.legislation.gov.uk/uksi/2002/1438/contents/made (2019). Accessed 19th Feb 2019.
43. NHS Digital. Independent Group Advising on the Release of Data. https://digital.nhs.uk/about-nhs-digital/corporate-information-and-documents/independent-group-advising-on-the-release-of-data (2019). Accessed 19th Feb 2019.
44. Whittlestone J, Nyrup R, A A, Dihal K, S. C. Ethical and societal implications of algorithms, data, and artifical intelligence: a roadmap for research. London: Nuffield Foundation; 2019.

BMJ Open

Liaison Psychiatry - Measurement and Evaluation of Service Types, Referral patterns and Outcomes - LP- MAESTRO: a protocol

Journal:	*BMJ Open*
Manuscript ID	bmjopen-2019-032179.R1
Article Type:	Protocol
Date Submitted by the Author:	09-Oct-2019
Complete List of Authors:	Smith, Chris; University of Leeds, Leeds Institute of Health Sciences<br>Hewison, Jenny; University of Leeds, Leeds Institute of Health Sciences<br>West, Robert; University of Leeds, Leeds Institute of Health Sciences<br>Guthrie, Elspeth; University of Leeds, Leeds Institute of Health Sciences<br>Trigwell, Peter; Leeds and York Partnership NHS Foundation Trust, National Inpatient Centre for Psychological Medicine<br>Crawford, Mike; Imperial College London, Centre for Psychiatry, Department of Medicine; Royal College of Psychiatrists, College Centre for Quality Improvement<br>Czoski Murray, Carolyn; University of Leeds, Leeds Institute of Health Sciences<br>Fossey, Matt; Anglia Ruskin University, Veterans and Families Institute for Military Research, Faculty of Health, Social Care and Education<br>Hulme, Claire; University of Exeter, University of Exeter Medical School<br>Tubeuf, Sandy; Universite catholique de Louvain<br>House, Allan; University of Leeds, Leeds Institute of Health Sciences
**Primary Subject Heading**:	Health services research
Secondary Subject Heading:	Mental health
Keywords:	routinely-collected NHS data, data linkage, care pathways, liaison psychiatry

SCHOLARONE™
Manuscripts

Liaison Psychiatry - Measurement and Evaluation of Service Types, Referral patterns and Outcomes - LP- MAESTRO: a protocol

Authors

Chris Smith (C.J.Smith@leeds.ac.uk)
Leeds Institute of Health Sciences (LIHS), University of Leeds, Leeds, UK

Jenny Hewison (J.Hewison@leeds.ac.uk)
Leeds Institute of Health Sciences (LIHS), University of Leeds, Leeds, UK

Robert M West (R.M.West@leeds.ac.uk)
Leeds Institute of Health Sciences (LIHS), University of Leeds, Leeds, UK

Elspeth Guthrie (E.A.Guthrie@leeds.ac.uk)
Leeds Institute of Health Sciences (LIHS), University of Leeds, Leeds, UK

Peter Trigwell (peter.trigwell@nhs.net)
National Inpatient Centre for Psychological Medicine, Leeds and York Partnership NHS Foundation Trust, Leeds, UK

Mike J Crawford (m.crawford@imperial.ac.uk)
College Centre for Quality Improvement, Royal College of Psychiatrists, London, UK
Centre for Psychiatry, Department of Medicine, Imperial College London, London, UK

Carolyn Czoski-Murray (C.J.CzoskiMurray@leeds.ac.uk)
Leeds Institute of Health Sciences (LIHS), University of Leeds, Leeds, UK

Matt Fossey (matt.fossey@anglia.ac.uk)
Veterans and Families Institute for Military Research, Faculty of Health, Social Care and Education, Anglia Ruskin University, Chelmsford, UK

Claire Hulme (C.T.Hulme@exeter.ac.uk)
University of Exeter Medical School, University of Exeter, Exeter, UK

Sandy Tubeuf (sandy.tubeuf@uclouvain.be)
Institute of Health and Society (IRSS), Université catholique de Louvain, Louvain-la-Neuve, Belgium

Allan House (A.O.House@leeds.ac.uk)
Leeds Institute of Health Sciences (LIHS), University of Leeds, Leeds, UK

Corresponding Author

Chris Smith (C.J.Smith@leeds.ac.uk)
Leeds Institute of Health Sciences (LIHS), University of Leeds, Leeds, UK

Liaison Psychiatry - Measurement and Evaluation of Service Types, Referral patterns and Outcomes - LP- MAESTRO: a protocol

Abstract

Introduction

We describe the protocol for a project that will use linkage of routinely-collected NHS data to answer a question about the nature and effectiveness of liaison psychiatry services in acute hospitals in England.

Methods and analysis

The project will use three data sources: i) Hospital Episode Statistics (HES), a database controlled by NHS Digital that contains patient data relating to emergency department, inpatient and outpatient episodes at hospitals in England; ii) ResearchOne, a research database controlled by The Phoenix Partnership (TPP) that contains patient data relating to primary care provided by organisations using the SystmOne clinical information system; and iii) clinical databases controlled by mental health trusts that contain patient data relating to care provided by liaison psychiatry services. We will link patient data from these sources to construct care pathways for patients who have been admitted to a particular hospital and determine those patients that have been seen by a liaison psychiatry service during their admission.

Patient care pathways will form the basis of a matched cohort design to test the effectiveness of liaison intervention. We will combine health care utilisation within care pathways using cost figures from national databases. We will compare the cost of each care pathway and the impact of a broad set of health-related outcomes to obtain preliminary estimates of cost-effectiveness for liaison psychiatry services. We will carry out an exploratory incremental cost-effectiveness analysis from a whole system perspective.

Ethics and dissemination

Individual patient consent will not be feasible for this study. Favourable ethical opinion has been obtained from the NHS Research Ethics Committee (North of Scotland) (REF: 16/NS/0025) for Work Stream 2 (Phase 1) of the LP-MAESTRO study. The Confidentiality Advisory Group at the Health Research Authority determined that Section 251 approval under Regulation 5 of the Health Service (Control of Patient Information) Regulations 2002 was not required for the study "*on the basis that there is no disclosure of patient identifiable data without consent*" (REF: 16/CAG/0037).

Results of the study will be published in academic journals in health services research and mental health. Details of the study methodology will also be published in an academic journal. Discussion papers will be authored for health service commissioners.

Keywords

Liaison psychiatry, routinely-collected NHS data, data linkage, care pathways

Strengths and limitations of this study

- Study designs based on the linkage of routinely-collected NHS data enable the generation of evidence regarding the cost-effectiveness and efficiency of health services, where alternative study designs such as RCTs or other individually-consented study designs would be infeasible.

- Whilst our study will evaluate the impact of acute inpatient hospital work carried out by liaison teams, and will not cover work undertaken in the Emergency Department, outpatient or primary care setting, the findings will be highly relevant, as previous claims for cost savings resulting from the implementation of liaison services have been primarily based on their inpatient hospital work.

- Study designs based on the linkage of routinely-collected NHS data present technical, ethical and legal challenges which must be considered from the start, including their implications for the validity and generalisability of insights and for project resources.

Introduction

Liaison psychiatry services provide assessment and treatment for people with co-existent physical and mental health problems (1-4). Such services are provided predominantly in the acute hospital setting in the United Kingdom, although more recently services have emerged to support the management of people with complex physical and mental health problems in primary as well as secondary care (5). Liaison psychiatry services have the potential to improve both the quality of care and overall outcomes for people with mental and physical health problems. There is also a suggestion that liaison psychiatry services in the acute hospital setting will produce cost savings by reducing length of stay, even though it is estimated that services see a small proportion (1-5%) of all patients admitted to acute beds (6-12). For these reasons, NHS England has invested in expanding liaison psychiatry services to acute hospitals (13, 14).

The research evidence has not been strong for the cost-effectiveness of liaison psychiatry *services* (15, 16) as opposed to evidence on the cost-effectiveness of some of the *individual interventions* used in those services. (17, 18). For that reason, more research is needed using robust designs derived from health services research. Claims for cost-effectiveness of liaison psychiatry go to the origins of the specialty before World War II. Individual components have been tested in Randomised Controlled Trials (RCTs), but there have been few attempts to judge the cost-effectiveness of whole services. Although it has been argued they can pay for themselves - the cost-offset debate of the 1980 and 1990s (19) - in truth the cost-effectiveness evidence for any liaison psychiatry service is limited. Holmes et al (20) identified only two RCTs, some smaller non-randomised studies include working age and older patients, and older and non-definitive work on cost-offsetting.

There are three main reasons why visiting the question now is timely.

First, cost pressures in the NHS and the emergence of commissioning led by Clinical Commissioning Groups (CCGs) will continue to lead to re-profiling of services and especially attempts to reduce unnecessary hospital costs. Work on developing and implementing risk stratification models are an illustration and it is interesting that many of these models identify co-morbid physical and mental health problems as a risk, both for increased healthcare costs and for the main driver of such costs which is unscheduled hospital admission. A study from Birmingham describing the evaluation of a rapid assessment, intervention and discharge service (previously widely known by the acronym RAID) was widely promoted and is leading to commissioning of similar services that will be hoped to reduce costs (21, 22). There were however substantial problems with that evaluation: it reported only on the first 9 months of delivery of a new service; it used a simple before and after design; it compared outcomes between referred and a matched group of non-referred patients in only 79 cases with minimal matching that cannot have dealt with indication bias; and much of the benefit was attributed to so-called indirect liaison cases who were not in fact seen by the service but assumed to benefit

by its existence in the hospital. Other 'RAID'-like services have also reported large savings in cost or reductions in hospital use following implementation (23, 24). So a key answer to the question of why this research is needed now is the pressing need to confirm or refute the very striking claims made for similar services, but using larger numbers and more robust research methods.

Second, there is a danger of losing sight of the other main functions of liaison psychiatry services, which don't exist only to reduce costs in the general hospital but which are aimed at improving the wellbeing of patients, some of whom are being treated entirely appropriately in the general hospital and happen to need mental health service input because of the complexity of their problems.

Third, and related to the second point above, service commissioning and provision will benefit from a more standardised approach to service descriptors. Without more detailed knowledge about how to define the service being commissioned and how to evaluate whether it is working to remit and to improve Health-Related Quality of Life (HRQL) for patients, there is a risk of enthusiastic commissioning of services that look superficially similar to each other and to a model reported as cost-effective, when in fact either at the time or over a period after commissioning there are differences in staffing or referral patterns that invalidate the original commissioning assumptions. An important function is served by studies that describe what patient groups actually receive - what sort of service and in what numbers.

This research is thus timely in exploring methods to evaluate the function and performance of liaison psychiatry services.

There are, however, challenges in conducting such research.

First, defining exposure to liaison psychiatry is difficult because there is substantial heterogeneity in the composition, purpose and size of liaison psychiatry services. For example, a recent survey conducted in England (25) found that just over half of the services provided a 24 hour 7-day service and only a third ran specialist outpatient clinics. Most of the services provided cover of acute hospital wards and emergency department and nearly all services were multidisciplinary, but staff mix varied such that about a third employed less than one fulltime equivalent of a consultant in psychiatry. Only a third of services had separate teams for older adults and adults of working age. These differences were not fully explained by variation in acute hospital characteristics. Also, the mechanism by which liaison psychiatry services might produce improved patient and organisational outcomes is unclear – for example, with some suggesting that the main benefit is by securing rapid discharge to community-based services, while others emphasise the specialist nature of shared in-patient management or of outpatient clinics provided by the service.

Second, defining service outcomes is difficult because there is also substantial heterogeneity in the patient populations seen in liaison services. For example, service outcomes (and performance indicators) will not be the same for somebody seen in the emergency department after an act of self-harm, an older person with post-operative agitated behavior seen on a surgical ward, or somebody with chronic unexplained pain referred from a pain clinic.

To evaluate the impact of liaison psychiatry services on the outcomes of patients in acute hospital settings, we therefore need to be able to do three things. *Patients* attending selected hospitals need to be characterized with respect to their physical and mental health. The prognosis for any given mental health problem is strongly influenced by the prior history of mental disorder, so the nature of the psychiatric problem needs to be described not just at the time of admission but in the preceding months. This longitudinal picture can only be determined reliably and for all patients in any sample by the use of routine data from primary care. Patient *healthcare contact* in both primary and secondary care services needs to be recorded. And *outcomes* need to be identified beyond the immediate spell in hospital. The heterogeneity of both patient population and service exposures requires a large study in terms of number of hospitals and patients.

In addition to these measurement problems, there is a challenge in choosing a robust research design. There have been several RCTs showing the effectiveness of individual components of liaison services, but an individually-randomised RCT of a whole service configuration would be impractical. The *heterogeneity of case mix* even in simpler services will require numbers beyond what could be reasonably recruited, and there are major challenges in obtaining a large representative sample when individual consent is required. Because many patients seen by liaison psychiatry services lack mental capacity at the time of service contact, an individually-consented study is not feasible and an individually-randomised RCT would not in any case answer the service-level question.

We also considered a cluster RCT of different liaison services. *Heterogeneity of service provision*, as identified in our national survey of services in England, would make such a study prohibitively large.

For these reasons, our view is that a large-scale observational study based upon analysis of *routinely-collected NHS data* and which is not predicated on individual patient consent is the best option.

Methods and analysis

Study design

Retrospective cohort design, comparing outcomes for patients admitted to hospital and seen by a liaison psychiatry service with two control groups – the first is a patient group who were admitted to the same

hospital in the same study period and matched on hospital inpatient and primary care data, but were not seen by the liaison psychiatry service. Because such a design could not entirely exclude confounding by indication, we will use a second matched patient group who had been admitted to a different hospital without a liaison psychiatry service in the same study period. This second group will also be matched using data from hospital and primary care records; however they will not have been selected on the basis of whether the responsible (acute hospital) consultant had made a decision about liaison psychiatry referral.

Aims and objectives

This study arises from a commissioning call by the UK's National Institute for Health Research and represents one component (Work Stream 2) of the wider project, LP-MAESTRO (26), which is designed to evaluate the cost-effectiveness and efficiency of liaison psychiatry service provision in the UK. The aim of the study described in this paper is to examine care pathways for the main target populations of liaison psychiatry services and estimate the outcomes and costs associated with care. Specifically, we will:

1. Use routinely-collected NHS data to identify patients referred to specific liaison psychiatry services and matched comparison patients who were not referred, with the aim of comparing within and between hospitals the effect of referral or non-referral of patients with similar characteristics.

2. Estimate the cost of the care pathways of patients referred to liaison psychiatry services, and the matched comparison patient groups, and the main determinants of those costs over 12 months after an index hospital episode.

We will characterise patients according to their contact with liaison psychiatry service, for example reason for referral, scheduled or urgent referral, and mental health diagnosis. We will determine those patients who were referred to liaison psychiatry services from acute (general hospital) sources, and a matched sample of cases from the same sources who were not referred. We will compare outcomes for certain marker conditions (such as mental-physical comorbidity, acute behavioural disturbance, cognitive impairment/dementia) in different liaison service configurations.

Data sources

Patient data that are relevant to the study are routinely-collected by clinicians and healthcare professionals to inform patient care. Such data are collected independently by the organisations that provide different services to patients, and only those variables that are required to fulfil the purpose(s) of these services are included. A limited number of standardised datasets are collected from organisations that provide care and are aggregated at national-level by organisations, such as NHS Digital. Some of these datasets (or

derivatives) are made available for research purposes. However, no single organisation can currently provide the data that is required for the study, so linkage is essential.

Hospital Episode Statistics (HES) (27) is a database controlled by NHS Digital that contains patient data relating to emergency department, inpatient and outpatient episodes for NHS hospitals in England. Episodes represent discrete periods of care under a particular consultant. Episodes can be combined into spells to represent the period of care from admission to discharge. HES is derived from the Commissioning Data Set (CDS) (28), which is supplied to NHS Digital by organisations that provide NHS services to facilitate monitoring and payment. Patients can opt-out of the inclusion of their *confidential patient information* in datasets which are made available by NHS Digital for purposes beyond care, such as HES, through the national opt-out programme (29). HES is an important source of data relating to health service interaction in secondary care. There are three significant limitations that are relevant to this study.

Firstly, referral to liaison psychiatry services cannot be reliably determined from HES. A new episode is not generated by such a referral - the patient remains within the care of the acute hospital consultant - and contact with a different consultant-led team in liaison psychiatry is not represented within an episode.

Secondly, it is suggested that mental health diagnoses recorded in routinely-collected NHS data, such as HES, exhibit variable accuracy with respect to the true diagnosis (30).

Thirdly, patient interactions with primary care services are not included in HES. Such data are required to match patients by defined characteristics and to determine the care delivered in primary care following a general hospital admission that leads to a liaison psychiatry referral.

To address the first limitation, clinical databases controlled by the mental health trusts that provide liaison psychiatry services will be used. Such databases contain patient data relating to care provided by liaison psychiatry services and can be used in conjunction with HES data to determine whether a patient was referred to a liaison psychiatry service during a hospital admission. The main challenges with the use of such databases are data quality and the heterogeneous processes by which data access is negotiated and administered within different organisations.

To address the second and third limitation, data relating to primary care is required for each patient. Although the Clinical Practice Research Datalink (CPRD) database (31) is widely used for primary care research in the United Kingdom, it has a major drawback for this study – limited numbers and geographical coverage of participating primary care organisations at the time of study design. Instead ResearchOne (32), a research database controlled by The Phoenix Partnership will be used. ResearchOne contains patient data relating to primary care provided by organisations using the SystmOne clinical information system (33).

SystmOne (34%) and EMIS (34) (56%) are the most prevalent clinical information systems used by organisations in general practice (35). Therefore, many of the patients with episodes in the HES data can be expected to have data relating to their primary care collected by organisations that use SystmOne. ResearchOne contains data for a subset of these patients – patients who: i) are registered to organisations that use SystmOne and have opted-in to participation in ResearchOne, and ii) have not individually opted-out of participation in ResearchOne. The main challenge with the use of ResearchOne is the inability to determine *a priori* the numbers and geographical coverage of organisations that provide primary care to patients attending the hospitals chosen for the study, and the resultant ability to match HES data to corresponding ResearchOne data for each patient.

Patient data from these three sources will be linked to construct patient care pathways that span primary and secondary care settings. Linkage will be undertaken by NHS Digital. Each data source will generate two unique references for each patient: i) a pseudonym - generated by applying a one-way cryptographic hash function (SHA-512) to an input that comprises a cryptographic salt and their NHS number, and ii) a source-specific identifier. For a patient with a given NHS number, each data source will generate the same pseudonym but a different source-specific identifier. Both the pseudonym and source-specific identifier generated for each patient will be specific to the study. Pseudonyms will be used by NHS Digital to: i) communicate to data sources those patients for whom data is required, and ii) generate mappings between the different source-specific identifiers for each patient. Data sources will provide the required patient data to the research team, including only the source-specific identifier as the unique reference for each patient. The mappings generated by NHS Digital will be provided to the research team and used to determine the data that relates to each patient across the data received from the different sources.

Data extraction

Based on the results from earlier stages of the LP MAESTRO project, we will identify at least two and up to six configurations that best represent patterns of liaison psychiatry service across England. Defining features of such configurations will include for example: staff mix, availability of specialist teams (for example age-related, self-harm), hours of service provided by the specialist team, and source of referrals (predominantly ED, predominantly ward, specialist services and so on). We will sample purposively to obtain 2-4 services of each type (depending upon availability). Data will be extracted for patients attending the hospitals with these service elements in a 1 year index period and also for patients attending hospitals identified as not having a liaison psychiatry service in the same period. Financial year 2013/2014 was selected as the index period as it represented the latest complete year for which data was available from NHS Digital at the time of study design. Numbers of A&E attendances and Inpatient admissions in the index period on which to estimate sample size are not publicly available at hospital-level. However, numbers are published by NHS Digital at trust-level (36, 37), where one trust operates one or more hospitals. These trust-level figures

provide an indicative upper bound on the A&E attendances and Inpatient admissions that can be expected at any hospital operated by that trust in a given year.

Relevant variables extracted for each patient from the sources will include demographic variables (e.g. age, carer support, Index of Multiple Deprivation – a measure of locality deprivation), clinical variables (e.g. diagnosis, medications) and health service utilisation variables (e.g. inpatient days, GP appointments, major procedures). One of the novelties of our approach is the use of variables obtained from primary and secondary care settings to tackle the substantial challenge that comes from indication bias; for example we will use variables obtained from ResearchOne to define healthcare utilisation in primary care for the year before referral (2013), as a way of ensuring that outcomes in the year after referral (2015) are not attributed to easily identifiable pre-existing characteristics (case complexity) that are confounded with likelihood of referral.

Patient care pathways for patients attending hospitals using liaison psychiatry services in each configuration will be constructed to provide a view of health and healthcare across both primary and secondary care. Pathways will be constructed for patients for a period of 12 months following their index (first) hospital admission in the index period. The cost of each pathway to the health care sector will then be calculated using national data sources (see below). We will adopt a whole system perspective in order to determine if there is an association between the configuration of liaison psychiatry services and health care utilisation by patients. Metrics including emergency admissions, occupied bed days and length of stay will be analysed by age band.

Data analysis

We will build a standard regression model to estimate the relation between health care utilisation and key variables capturing the configuration of liaison psychiatry services. The dependent variable in this model will therefore be the total costs of any identified health care utilisation derived from factors such as inpatient days, readmission rates, ED attendances and GP visits combined with reference costs. These reference costs will be valued using national sources, including the Department of Health Reference Costs (38) and Personal Social Services Research Institute (PSSRU) Costs for Health and Social Care. (39). Where these are not available, local costs will be assigned. We will choose the most appropriate base year for the analysis and adjust appropriately for the effects of inflation across years. The quantum of the liaison service provision will be captured by already collected data related to structure and process; for example, staffing levels and contact time after referral. We will adjust for referral indication bias, either by matching for co-variates or by propensity scoring.

Sample size is difficult to estimate because we have so little available data on outcomes for different service types and different patient groups. Suppose we identify six main service configurations and recruit two liaison services for each (total n=12). For less common conditions we might expect to see one referral per week per service = 100 in total in the year. For more common conditions we might perhaps see one referral per day or 600-800 in the year. These numbers will allow us to estimate the costs and cost-effectiveness of liaison services with substantially greater precision than has been achieved to date – for example by the RAID evaluation.

The way in which components of general hospital, general mental health and liaison services interact with each other is complex and a key part of the project will be to determine how to capture this complexity into a set of measures for inclusion in the model.

We (CH, ST) will carry out exploratory incremental cost effectiveness analyses using decision analysis modelling. The model will rely on the retrospectively estimated healthcare costs of alternative care pathways and the characteristics at hospital, service and patient-level. Given the nature of the data available, the absence of measures such as Quality of Life measures and the heterogeneous nature of the population, we will explore the use of a range of variables to assess effectiveness and evaluate the costs per length of stay, per re-admission and per life years lost. The health economics analyses will be informed by earlier work packages in the LP-MAESTRO project. We will however also follow the guidance from the National Institute for Clinical Excellence (NICE) for methods for technology appraisal (40). However, whilst it is clear that some aspects of an exploratory model (or indeed models) may be specified in advance, for example, the perspective of the economic evaluation which will be the health service provider and the comparator which will be usual care, other aspects will be dependent upon the shape of the services and the populations they engage with.

At this point we are unable to specify the time horizon of the decision analysis model evaluating the long-term cost-effectiveness of liaison services. We will look to a long-term model and use NICE recommended discount rates for costs. The model itself is likely to be Markov or semi-Markov. Sensitivity analyses will be undertaken in line with those recommended for this type of modelling (41). Presentation of the analysis or analyses will include incremental cost-effectiveness ratios (ICERs), cost-effectiveness acceptability curves and net monetary benefit estimates. In addition, we will undertake a value of information (VOI) analysis (42).

Patient and public involvement

The study has a Patient and Public Involvement (PPI) representative on the Study Steering Committee that oversees the management of the research.

Ethics and dissemination

The study is funded by the National Institute of Health Research under the Health Service and Delivery Research programme (REF: 13/58/08) and is sponsored by the University of Leeds. The study is based at the Leeds Institute of Health Sciences within the University of Leeds and will use the Information Governance Toolkit (IGT) compliant infrastructure at the Leeds Institute of Clinical Trials Research (REF: ECC0010).

To simplify the documentation provided to underpin ethics and governance processes, the study has been partitioned into distinct phases. Each phase is characterized by the use of a specific combination of data sources to construct care pathways for patients attending a particular hospital or set of hospitals. Phase 1 is characterized by the use of data from HES and ResearchOne *only* to construct care pathways for patients attending hospitals *without* a liaison psychiatry service. A summary of the ethics and governance processes undertaken for Phase 1 is provided below.

As described in the Introduction section, individual patient consent will not be feasible for this study.

Phase 1 was submitted to the NHS Research Ethics Committee (North of Scotland) on 23rd February 2016 (REF: 16/NS/0025). The application was reviewed in a meeting held on the 10th March 2016 and received a favorable ethical opinion on 15th March 2016. Favorable ethical opinion was contingent on obtaining management permission from the hospitals whose patients were to be included in the HES data. Management permission was received for 8 of the 11 hospitals by a stated deadline (15th December 2016) and Phase 1 proceeded based on the use of HES data from these 8 hospitals only.

Phase 1 was also submitted to the Confidentiality Advisory Group (CAG) (43) at the Health Research Authority on 23rd February 2016 (REF: 16/CAG/0037) to determine whether the study required Section 251 support under Regulation 5 of the Health Service (Control of Patient Information) Regulations 2002 (44). The application was reviewed in a meeting on 21st April 2016 and was deferred pending the receipt of further information in relation to the linkage process from the research team. Further information was supplied and CAG provided a decision on 19th July 2016 that Section 251 support was not required "*on the basis that there is no disclosure of patient identifiable data without consent*".

Based on favorable ethical opinion and a decision from CAG that Section 251 support was not required, approval in principle for Phase 1 was provided by TPP on 11th October 2016. Data requests were submitted to NHS Digital on 16th December 2016 (REF: NIC-77953) and TPP on 22nd February 2017. Supporting evidence documents were provided with these requests, which included confirmation of favorable ethical opinion, confirmation of the CAG decision on Section 251 support, and details of the technical and organisational safeguards in place at the data controller and processors. Organisational safeguards included

a Data Processing Agreement established between University of Leeds and TPP to cover the data processing activities within Phase 1. The application was reviewed by the Independent Group Advising on the Release of Data (IGARD) (45) at NHS Digital on 20th March 2018. Further information was requested from the project team by IGARD, which was subsequently supplied, and a recommendation to approve the application was provided in a meeting on 26th April 2018. A Data Sharing Agreement (DSA) was established between the University of Leeds and NHS Digital for Phase 1 on 26th April 2018, which was underpinned by the pre-existing Data Sharing Framework Contact between the University of Leeds and NHS Digital (REF: CON-315426-K3W7R).

Data were supplied by NHS Digital on 16th November 2018 and the remaining data from NHS Digital and TPP are currently awaited.

Results of the study will be published in academic journals in health services research and mental health. Details of the study methodology will also be published in an academic journal. Discussion papers will be authored for health service commissioners.

Discussion

Studies based on the linkage of routinely-collected NHS data enable the generation of evidence regarding the cost-effectiveness and efficiency of health services, where alternative study designs such as RCTs or other individually-consented study designs would be infeasible. Health service commissioners can be provided with robust evidence to underpin decisions regarding health services and interventions – in this case relating to the cost-effectiveness and efficiency of different liaison psychiatry configurations for specified target populations – where previously there may have been limited or no evidence. The findings of this study will evaluate the impact of acute inpatient hospital work carried out by liaison teams, and does not cover work undertaken in the Emergency Department, outpatient or primary care setting. The findings, however, will be highly relevant, as previous claims for cost savings resulting from the implementation of liaison services have been primarily based on their inpatient hospital work.

Studies such as this present technical, ethical and legal challenges. Study designs should consider such challenges from the start, including their implications for the validity and generalisability of insights and for project resources. Experience from LP-MAESTRO demonstrates that significant resources are required to design and communicate a research protocol in the manner that satisfies the different project stakeholders, including research ethics committees, data controllers, regulatory bodies and data access committees. Different stakeholders are focused on their specific remit and require communication of the research protocol in accordance with that remit. Moreover, different stakeholders may comprise of decision-makers

from different disciplines and require the research protocol to be communicated at differing levels of abstraction to ensure adequate comprehension. This issue of communication between disciplines and the potential for misinterpretation is highlighted in a recent Nuffield foundation report (46).

The project described here is both technically feasible and consistent with current legislative and ethical frameworks applicable to the use of health data for research purposes. The main practical challenges reside in the communication with, negotiation between, and coordination of different stakeholders as outlined above.

There are potential limitations to the findings: it may not be easy to derive clearly discrete configurations of service from routinely available data; although matching via primary care records will allow more precision than can be managed from routine hospital data, the data available for matching will still be limited and not standardised across records; although a multi-site study will generate substantial numbers (a typical liaison service will make 2000+ new contacts a year) sample sizes for particular subgroups of patients may be too small for meaningful analysis.

List of abbreviations

RCTs: Randomised Controlled Trials
CCGs: Clinical Commissioning Groups
HRQL: Health-Related Quality of Life
HES: Hospital Episode Statistics
CDS: Commissioning Data Set
CPRD: Clinical Practice Research Datalink
PSSRU: Personal Social Services Research Institute
NICE: National Institute for Clinical Excellence
VOI: Value of Information
IGT: Information Governance Toolkit
CAG: Confidentiality Advisory Group
IGARD: Independent Group Advising on the Release of Data
DSA: Data Sharing Agreement
TPP: The Phoenix Partnership

Competing interests

CS is Director of PrivacyForge Limited. All other authors declare that they have no competing interests.

Funding

This project is funded by the National Institute for Health Research (NIHR) Health Services and Delivery Research (HS&DR) programme (project reference 13/58/08).

Authors' contributions

CS, JH, RW, EG, PT, MC, CCM, MF, CH, ST, and AH contributed to the design of the study protocol. AH, JH and CS authored the manuscript. CS, JH, RW, EG, PT, MC, CCM, MF, CH, ST, and AH contributed to the revision of the manuscript and approved the final version.

Acknowledgements

The views expressed are those of the author(s) and not necessarily those of the National Institute for Health Research (NIHR) or the Department of Health and Social Care.

References

1. Aitken P, Lloyd G, Mayou R, Bass C, Sharpe M. A history of liaison psychiatry in the UK. BJPsych Bull. 2016;40(4): 199-203.
2. Lipowski Z. Consultation-liaison psychiatry: the first half century. General hospital psychiatry. 1986;8(5): 305-15.
3. Mayou R. The history of general hospital psychiatry. Br J Psychiatry. 1989;155: 764-76.
4. Schwab JJ. Consultation-liaison psychiatry: a historical overview. Psychosomatics. 1989;30(3): 245-54.
5. Bestall J, Siddiqi N, Heywood-Everett S, Freeman C, Carder P, James M, et al. New models of care: a liaison psychiatry service for medically unexplained symptoms and frequent attenders in primary care. The Psychiatrist: Bulletin of the Royal College of Psychiatrists. 2017;41(6): 340-4.
6. Huyse, F.J., et al. European consultation-liaison services and their user populations: the European Consultation-Liaison Workgroup Collaborative Study. Psychosomatics. 2000;41(4): 330-8.
7. Diefenbacher, A., Implementation of a psychiatric consultation service: a single-site observational study over a 1-year-period. Psychosomatics. 2001;42(5): 404-10.
8. Christodoulou, C., et al. Transfers to psychiatry through the consultation-liaison psychiatry service: 11 years of experience. Ann Gen Psychiatry, 2008. 7.
9. Su, J.A., et al. Changes in consultation-liaison psychiatry in the first five years of opearation of a newly-opened hospital. Chang Gung Med J. 2010;33(3): 292-300.
10. Sanchez-Gonzalez, R., et al. Clinical features of a sample of inpatients with adjustment disorder referred to a consultation-liaison psychiatry service over 10years. Gen Hosp Psychiatry. 2018;55: 98-99.
11. Sanchez-Gonzalez, R., et al. Clinical profile of inpatients referred to a consultation-liaison psychiatry service: an observational study assessing changes over a 10-yearperiod. Actas Esp Psiquiatr, 2018;46(2): 58-67.
12. Gala, C., et al. A multicenter investigation of consultation-liaison psychiatry in Italy. Gen Hosp Psychiatry. 1999;21(4): 310-7.
13. NHS England. Five Year Forward View. London: NHS England; 2014.
14. NHS England. Urgent and emergency mental health liaison in acute hospitals (adult and older adults): Supporting documentation for transformation funding selection. London: NHS England; 2016.
15. de Araujo Andreoli PB, de Albuquerque Citero V, de Jesus Mari JJP. A systematic review of studies of the cost-effectiveness of mental health consultation-liaison interventions in general hospitals. Psychosomatics. 2003;44(6): 499-507.
16. Hosaka T, Aoki T, Watanabe T, Okuyama T, Kurosawa HJP. General hospital psychiatry from the perspective of medical economics. Psychiatry and Clinical Neurosciences. 1999;53(4): 449-53.

17. Ruddy R, House A. Meta-review of high-quality systematic reviews of interventions in key areas of liaison psychiatry. Br J Psychiatry. 2005;187: 109-20.
18. Wood R, Wand APF. The effectiveness of consultation-liaison psychiatry in the general hospital setting: A systematic review. Journal of Psychosomatic Research. 2014;76(3): 175-92.
19. Strain JJ, Hammer JS, Fulop G. APM task force on psychosocial interventions in the general hospital inpatient setting: a review of cost-offset studies. Psychosomatics. 1994;35(3) :253-62.
20. Holmes A, Handrinos D, Theologus E, Salzberg M. Service use in consultation-liaison psychiatry: guidelines for baseline staffing. Australasian Psychiatry. 2011;19(3): 254-8.
21. Parsonage M, Fossey M. Economic Evaluation of a Liaison Psychiatry Service. London: Centre for Mental Health; 2011.
22. Tadros G SR, Kingston P, Mustafa N, Johnson E, Pannell R, Hashmi M. Impact of an integrated rapid response psychiatric liaison team on quality improvement and cost savings: the Birmingham RAID model. The Psychiatrist. 2013;37: 4-10.
23. Becker L, S.R., Hardy R, Pilling S. The RAID Model of Liaison Psychiatry: Report on the Evaluation of Four Pilot Services in East London. London: UCL Partners Academic Health Science Partnership; 2016.
24. Opmeer, B.C., et al., Extending the liaison psychiatry service in a large hospital in the UK: a before and after evaluation of the economic impact and patient care following ED attendances for self-harm. BMJ Open. 2017;7(8).
25. Walker A, Barrett JR, Lee W, West RM, Guthrie E, Trigwell P, et al. Organisation and delivery of liaison psychiatry services in general hospitals in England: results of a national survey. BMJ Open. 2018;8(8).
26. National Institute for Health Research (NIHR). Liaison Psychiatry: Measurement And Evaluation of Service Types, Referral patterns and Outcomes (LP-MAESTRO). https://www.journalslibrary.nihr.ac.uk/programmes/hsdr/135808 (2019). Accessed 19th Feb 2019.
27. NHS Digital. Hospital Episode Statistics (HES). https://digital.nhs.uk/data-and-information/data-tools-and-services/data-services/hospital-episode-statistics (2019). Accessed 19th Feb 2019.
28. NHS Digital. Commissioning Datasets. https://digital.nhs.uk/data-and-information/data-collections-and-data-sets/data-sets/commissioning-data-sets (2019). Accessed 11th Apr 2019.
29. NHS Digital. National Opt-Out Programme. https://digital.nhs.uk/services/national-data-opt-out-programme (2019). Accessed 19th Feb 2019.
30. Davis KA, Sudlow CL, Hotopf MJBp. Can mental health diagnoses in administrative data be used for research? A systematic review of the accuracy of routinely collected diagnoses. BMC Psychiatry. 2016;16(1):263.
31. Clinical Practice Research Datalink. https://www.cprd.com (2019). Accessed 19th Feb 2019.
32. The Phoenix Partnership. ResearchOne. http://www.researchone.org (2019). Accessed 19th Feb 2019.

33. The Phoenix Partnership. SystmOne. https://www.tpp-uk.com/products/systmone (2019). Accessed 19th Feb 2019.
34. Egton Medical Information Systems. EMIS Web. https://www.emishealth.com/products/emis-web/ (2019). Accessed 19th Feb 2019.
35. Kontopantelis E, Stevens RJ, Helms PJ, Edwards D, Doran T, Ashcroft DMJBo. Spatial distribution of clinical computer systems in primary care in England in 2016 and implications for primary care electronic medical record databases: a cross-sectional population study. BMJ Open. 2018;8(2).
36. NHS Digital. Accident and Emergency Attendances in England - 2013-14. https://digital.nhs.uk/data-and-information/publications/statistical/hospital-accident--emergency-activity/2013-14. Accessed 11th September 2019.
37. NHS Digital. Hospital Episode Statistics, Admitted Patient Care, England - 2013-14. https://digital.nhs.uk/data-and-information/publications/statistical/hospital-admitted-patient-care-activity/hospital-episode-statistics-admitted-patient-care-england-2013-14. Accessed 11th September 2019.
38. Department of Health. Reference costs 2015–16. London, UK: NHS England, 2016.
39. Curtis, L. & Burns, A. (2018) Unit Costs of Health and Social Care 2018, Personal Social Services Research Unit, University of Kent, Canterbury.
40. National Institute for Health and Care Excellence. Guide to the methods of technology appraisal. London: NICE, 2013.
41. Briggs R, Sculpher M, Claxton K. Decision Modelling for Health Economic Evaluation. Oxford, UK: Oxford University Press; 2006.
42. Petrou S, Gray A. Economic evaluation using decision analytical modelling: decision, conduct, analysis, and reporting. BMJ 2011;342.
43. Health Research Authority. Confidentiality Advisory Group. https://www.hra.nhs.uk/approvals-amendments/what-approvals-do-i-need/confidentiality-advisory-group (2019). Accessed 19th Feb 2019.
44. HM Government. The Health Service (Control of Patient Information) Regulations 2002. http://www.legislation.gov.uk/uksi/2002/1438/contents/made (2019). Accessed 19th Feb 2019.
45. NHS Digital. Independent Group Advising on the Release of Data. https://digital.nhs.uk/about-nhs-digital/corporate-information-and-documents/independent-group-advising-on-the-release-of-data (2019). Accessed 19th Feb 2019.
46. Whittlestone J, Nyrup R, A A, Dihal K, S. C. Ethical and societal implications of algorithms, data, and artifical intelligence: a roadmap for research. London: Nuffield Foundation; 2019.
